# Household Transmission of Rotavirus in a Community with Rotavirus Vaccination in Quininde, Ecuador

**DOI:** 10.1371/journal.pone.0067763

**Published:** 2013-07-09

**Authors:** Ben Lopman, Yosselin Vicuña, Fabian Salazar, Nely Broncano, Matthew D. Esona, Carlos Sandoval, Nicole Gregoricus, Michael D. Bowen, Daniel Payne, Martiza Vaca, Martha Chico, Umesh Parashar, Philip J. Cooper

**Affiliations:** 1 Division of Viral Diseases, Centers for Disease Control and Prevention; Atlanta, Georgia, United States of America; 2 Fundación Ecuatoriana Para Investigación en Salud, Quinindé, Esmeraldas Province, Ecuador; 3 Universidad de San Francisco de Quito; Quito, Ecuador; 4 Department of Parasitology, Liverpool School of Tropical Medicine; Liverpool, United Kingdom; National Institutes of Health, United States of America

## Abstract

**Background:**

We studied the transmission of rotavirus infection in households in peri-urban Ecuador in the vaccination era.

**Methods:**

Stool samples were collected from household contacts of child rotavirus cases, diarrhea controls and healthy controls following presentation of the index child to health facilities. Rotavirus infection status of contacts was determined by RT-qPCR. We examined factors associated with transmissibility (index-case characteristics) and susceptibility (household-contact characteristics).

**Results:**

Amongst cases, diarrhea controls and healthy control household contacts, infection attack rates (iAR) were 55%, 8% and 2%, (n = 137, 130, 137) respectively. iARs were higher from index cases with vomiting, and amongst siblings. Disease ARs were higher when the index child was <18 months and had vomiting, with household contact <10 years and those sharing a room with the index case being more susceptible. We found no evidence of asymptomatic infections leading to disease transmission.

**Conclusion:**

Transmission rates of rotavirus are high in households with an infected child, while background infections are rare. We have identified factors associated with transmission (vomiting/young age of index case) and susceptibility (young age/sharing a room/being a sibling of the index case). Vaccination may lead to indirect benefits by averting episodes or reducing symptoms in vaccinees.

## Introduction

Rotavirus is the most common cause of severe pediatric gastroenteritis and is estimated to cause approximately 450,000 deaths globally per year [1]. Rotavirus vaccines have been shown to prevent severe diarrheal disease in a range of settings, although their efficacy is reduced in lower socioeconomic settings, particularly in poor populations living in the tropics [2]. Infection with rotavirus can occur throughout life, although symptomatic illness is mainly restricted to children under five with severe and life-threatening disease occurring before two years of age. [1] Almost all unvaccinated children will be infected with rotavirus in the first two years of life [3], [4]. Primary infections are protective against subsequent disease, with severe disease becoming rare in secondary and subsequent infections [4]. Viral shedding is highly correlated with severity of disease and disease severity is inversely associated with number of previous infections. By mimicking the first and one or two subsequent infections, rotavirus vaccines are particularly effective against severe disease, relative to all episodes of rotavirus gastroenteritis [5], [6]. It follows that rotavirus vaccination has the potential to reduce levels of wild virus shedding and reduce levels of environmental contamination with virus. However, rotavirus is highly transmissible, with a very low infectious dose; to reduce the probability of onward transmission, shedding may have to be reduced below some threshold level.

Aside from the direct protective effects offered to children immunized against rotavirus, there may also be indirect effects of vaccination. Indeed, the magnitude of vaccine impacts in some settings suggest that vaccination may interrupt transmission and provide indirect protection [7]. Recent post-licensure studies in the United States and Australia have reported reductions of disease in unvaccinated groups, including children and adults too old to have been vaccinated [8], [9]. This suggests the prime importance of young children in rotavirus transmission. Much remains unknown about the transmission process of rotavirus and other enteric virus infections and, therefore, how vaccination could affect their transmission.

It is unknown whether vaccination will reduce infectiousness and thereby interrupt transmission, and afford indirect benefits to unvaccinated individuals in vaccinated populations in developing countries. We aimed to study the transmission of rotavirus in households following the presentation of a child to health services for rotavirus gastroenteritis in a vaccinated population in rural Ecuador.

## Methods

### Recruitment of Index Cases/Controls

From Feb 2011 to May 2012, children aged 6 to 59 months presenting with diarrhea to the Hospital Padre Alberto Buffoni (HPAB) in Quininde, Ecuador and surrounding family health clinics were considered for enrolment. Diarrhea was defined as three or more liquid or semi-liquid stools in 24 hours, with duration less than 14 days before consultation with the clinic/hospital. When a child presented with diarrhea, a nurse collected a fecal specimen and clinical data. Fecal specimens were tested for presence of rotavirus antigen by enzyme immunoassay (EIA). Children testing positive for rotavirus by EIA were defined as *cases*. Two comparison groups were recruited: (1) children who presented with diarrhea but tested negative for rotavirus by EIA (henceforth referred to as *diarrhea controls*) and healthy children without diarrhea, vomiting or hospitalization for any cause 14 days prior to recruitment and members of an ongoing birth cohort study who presented to the HPAB for routine follow-ups [10] (henceforth referred to as *healthy controls*). Both groups of controls were matched for age (+/−6 months for cases under age 3 and +/−1 year for cases aged 3 to 4 years).

After cases of rotavirus were detected, fecal samples were requested from all adult and child household members of the case during a household visit. Households were visited twice. At the first visit, the head of the household was informed about the household study, given consent procedures, and specimen collection pots were left for each household member. Henceforth, we refer to the three household populations as case households, diarrhea control households, and healthy control households, based on the disease and infections status of the index case. Specimen collection was requested as the date of onset of diarrhea in the index child (case or control) +7 days or as near as possible (5 to 9 days after onset). An adult representative of the household was interviewed for information on demographics, date of specimen collection and presence of diarrhea in the 10 days before and 10 days after the onset of diarrhea in the index case. For healthy control households, specimens were taken within one day of the household visit and information was collected on any symptoms in the 10 days before the index date of recruitment.

This study and consent procedures were reviewed and approved by the Bioethics Committee of the Universidad de San Francisco de Quito. All recruited household members were explained the premise of the project and were asked to provide written informed consent prior to enrollment in the study. A parent or guardian was asked to provide written consent on behalf of minors in the household.

### Specimens and Testing

Specimens taken following episodes of diarrhea in index cases were tested for rotavirus using the Prospect™ Rotavirus Test (Oxoid Diagnostics Ltd, United Kingdom). This test was used to initially determine if the diarrhea presentations in the clinic/hospital were cases (rotavirus-positive) or diarrhea controls (rotavirus negative). The Prospect™ kit and other validated enzyme immunoassays are calibrated to detect rotavirus at levels considered to cause disease. The majority of rotavirus infections in household members were expected to be asymptomatic. In order to detect asymptomatic infections, real time reverse transcription and quantitative polymerase chain reaction (RT-qPCR) was used, due to its much greater analytic sensitivity [11]–[13]. Note that stools of all symptomatic index children (cases and diarrhea controls) were tested by RT-qPCR (following EIA), while household contact were only tested by RT-qPCR.

Remaining specimens were stored at −20°C for conventional RT-PCR and genotyping. Genotyping by RT-PCR was performed on positive samples to confirm transmission within a household (i.e. to confirm that secondary cases are infected with the same type as the primary cases in the household).

### Diagnostic and Genotyping Methods

For EIA or RT-qPCR confirmed positive samples, total RNA was extracted by using the MagMax Viral RNA Isolation kit (Life Technologies, New York, NY) on the automated KingFisher extraction platform (Thermo Electron Corporation, Vantaa, Finland).

G-type (VP7) and P-type (VP4) genotyping were carried out following previously described 2-step amplification methods [14], [15] with modification on a GeneAMP PCR System 9700 thermocycler (Applied Biosystems, Foster City, CA). VP7 and VP4 were amplified by RT-PCR using consensus primers 9Con1-L/VP7R [14] and Con3/Con2, [15] respectively. The RT-PCR step was modified to use the One-Step RT-PCR kit (Qiagen, Inc., Valencia, CA) on a GeneAMP PCR System 9700 thermocycler (Applied Biosystems, Foster City, CA). In this procedure, the extracted dsRNA was denatured at 97°C for 5 min and RT-PCR was carried out using a One Step RT-PCR kit (Qiagen, Inc., Valencia, CA) according to manufacturer’s instructions. Reverse transcription (RT) of each gene was carried out for 30 min at 42°C, followed by 15 min at 95°C to inactivate the reverse transcriptase and activate the Taq polymerase. The cDNA was then subjected to 35 cycles of PCR in a GeneAmp PCR System 9700 thermal cycler (Applied Biosystems, Inc., Foster City, CA) using the following conditions: 30 sec at 94°C; 30 sec at 42°C; 45 sec at 72°C, followed by a 7 min final extension at 72°C. Genotyping PCR was performed as described previously. [14], [15] G-typing used primer 9Con1-L in combination with primers 9T1–1, 9T1-Dg, 9T-2, 9T-3P, 9T-4, and 9T-9B [14] and P-typing used primer con3 in combination with primers 1T-1, 1T1-VN, 2T-1, 3T-1, 4T-1, 5T-1, and 1T1-Wa [15]. Genotyping reactions were analyzed by electrophoresis on a 3% agarose gel using a 2∶1 ratio of NuSieve GTG: SeaPlaque (FMC Bioproducts, Rockland, ME). The G- and P-types obtained were classified according to the system described by Estes and Cohen [16]. G1 viruses were examined by specific primers to determine if they were vaccine (Rotarix) strains.

### Nucleotide Sequencing and Sequence Analysis

A selected number of isolates from the same household with the same genotyping results were subjected to sequencing for confirmation. The same consensus primer pair con3/con2 for VP4 gene and 9Con1-L/VP7R for VP7 gene were used to generate amplicons for the sequence reaction. Analysis of RT-PCR reactions by gel electrophoresis, amplicon purification, and DNA sequencing was carried out as described previously [17]. Forward and reverse sequences were assembled using Sequencher software versions 4.8 (Gene Codes Corporation, Inc, Ann Arbor, MI). The consensus sequences obtained were compared with existing rotavirus sequences in the GenBank database using the BLASTN program at the National Center for Biotechnology Information website (available at: http://www.ncbi.nlm.gov/BLAST/).

*Infection* amongst household contacts was defined as rotavirus detected by RT-qPCR in stool at the time of specimen collection; *disease* was defined as diarrheal disease symptoms within 10 days of onset in the index case. We also report disease attack rates based on diarrheal disease symptoms and detection of rotavirus by RT-qPCR, since it is possible that some of the PCR-negative individuals were symptomatic due to something other than rotavirus. All new viral sequence data has been deposited in GenBank; strain names and accession numbers can be found in File S1. Accession numbers for submitted strains.

### Statistical Analysis

We investigated potential risk factors for transmissibility (characteristics of cases) and susceptibility (characteristics of household contacts). Only household contacts of rotavirus index cases (n = 137) were included in the statistical analysis of transmission. We calculated the infection and disease attack rates and fit logistic regression models to estimate the odds ratio separately for each outcome. Initially, we attempted to fit random effects logistic regression models in order to explicitly account for correlation in outcomes with households. However, a number of bivariate models could not achieve convergence, so instead of fitting a random effects model, robust standard errors were calculated, treating each household as a cluster. First, bivariate models were fitted for each potential transmissibility and susceptibility factor. Separate models were fitted, with infection and disease as separate binary outcomes.

Then, we attempted to control for confounding by developing multivariable regression models. First, two separate were constructed by inclusion of all variables with p<0.20 in univariable analysis. The first model included only case (transmissibility) variables; the second included only contact (susceptibility) variables Then, using the same criteria, final models were fit with both transmissibility and susceptibility variables. Only one of a set of highly collinear variables (e.g. vomiting duration and severity score) was retained in multivariable models. Analyses were conducted in Stata 12.0 (STATA Corp, College Station, Tx).

## Results

**Figure 1:**
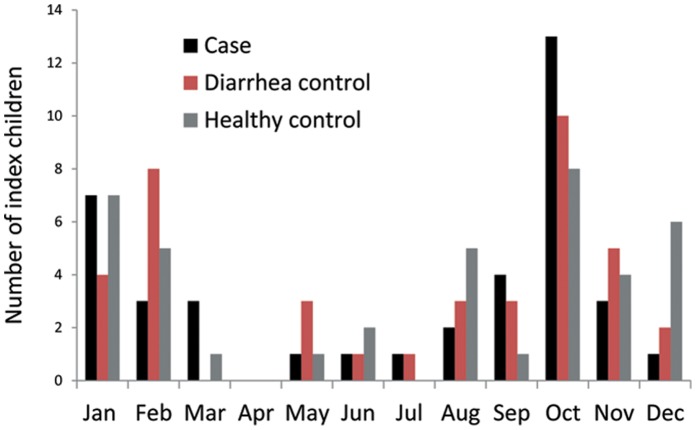
Calendar month distribution of index cases (n = 39) diarrhea controls (n = 40) and healthy controls (n = 40).

A total of 214 children were screened for rotavirus, 40 (19%) of which were positive by EIA and were recruited as index cases. One EIA positive subject could not be confirmed by RT-PCR so was excluded from subsequent analysis. 40 (22%) of the 175 rotavirus EIA-negative children with diarrhea were recruited as diarrhea controls; 40 well-child households were recruited as healthy controls. Case, diarrhea control and healthy control index children were similar in terms of month of enrolment (chi-squared p-value = 0.48, Figure 1) and median age (23, 24 and 26 month; t-test p-values = 0.49 & 0.27, respectively).

There were 197, 158 and 163 household members in case, diarrhea control and healthy control households, 137 (70%), 130 (82%) and 137 (84%) of whom provided a stool specimen, respectively. Case households (mean = 4.9 members) were somewhat larger than diarrhea control (mean = 4.0 members; t-test p-value = 0.09) and healthy control households (mean = 4.1 members; t-test p-value = 0.15).

### Infection

**Figure 2:**
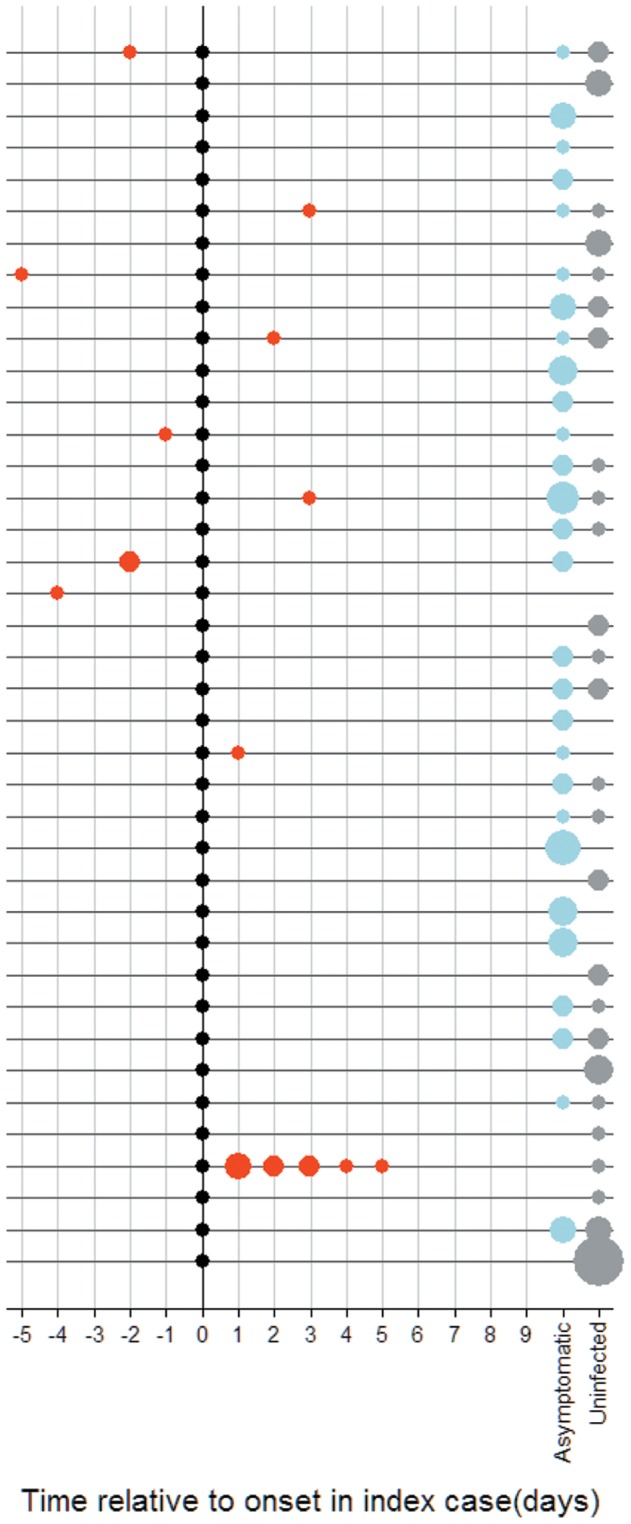
Infection and disease households with a rotavirus index case. Each line represents infection and disease events in one household. Index cases are plotted in black (n = 39). Other cases of rotavirus gastroenteritis in the household are plotted in red and are plotted on the time axis in terms of time of onset relative to time of onset of the index case. Household contacts with asymptomatic infection are plotted in blue (off the time scale, since we cannot know at what time they become infected) and contacts remaining uninfected are plotted in grey. The size of the points are relative to the number of individuals with a given outcome; for reference the size of the index cases (black dots) represents a single individual.

Infection was detected by RT-qPCR in 55% (76/137) of case household contacts, 7.6% (10/130) of diarrhea control contacts and 2.2% (3/137) healthy control contacts (Figure 2).

**Figure 3:**
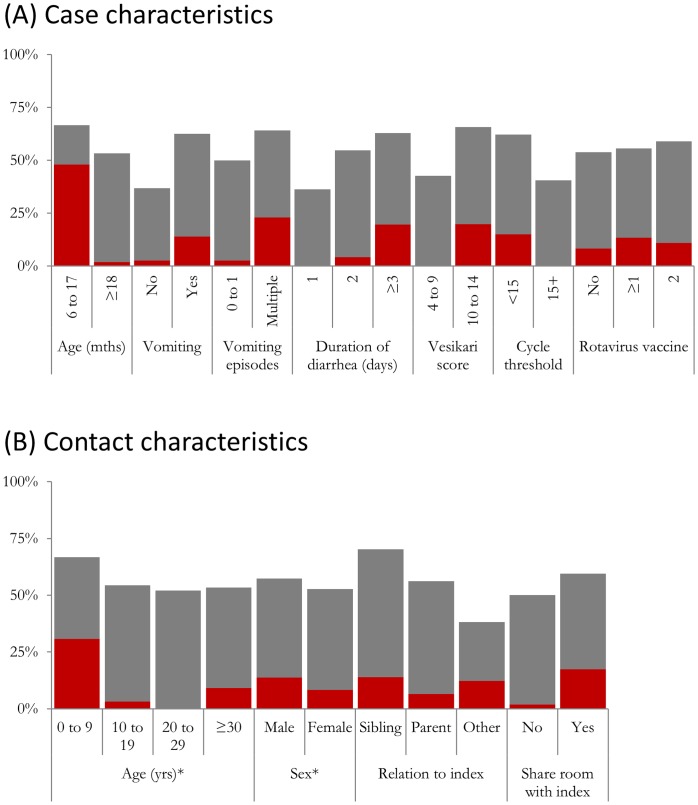
Infection (grey) and disease (red) attack rates amongst household contacts of index cases for (A) case characteristics and (B) contact characteristics.

The effects of index case characteristics (i.e. transmissibility) (i.e. index case characteristics) and contact characteristics (i.e. susceptibility) on infection attack rates are shown in Table 1. Regarding transmissibility, infection attack rates were non-significantly higher from younger children (67% from children 6 to 17 months compared to 53% from children ≥18 months); those who had vomiting (63% compared to 37%; OR = 2.87, p = 0.099) and those with Vesikari scores ≥10 (66% compared to 43%; OR = 2.58, p = 0.086), although these differences were of borderline statistical significance (Table 1 and Figure 3). Children with higher cycle threshold (Ct) values (indicative of lower viral load) were more likely to transmit though not at a level of statistical significance (62% with Ct <15; 40% with Ct was ≥15; OR = 0.41, p = 0.177). We did not find evidence of reduced transmissibility associated with a history of rotavirus vaccine, though study power was limited due to high vaccine coverage; 85% and 87% of index cases had one or two doses of vaccine, respectively.

**Table 1:** Infection attack rates amongst household contacts by their own characteristics and index case characteristics.

					Univariate		Multivariate(other case or contact characteristics)		Multivariate(case and contact characteristics)	
			n	Infection (%)	OR (95% CI)	p	OR (95% CI)	p	OR (95% CI)	p
**Contact characteristics**										
	Age (years)*	0 to 9	30	20 (67%)	1.75 (0.64–4.79)	0.276				
		10 to 19	35	19 (54%)	1.04 (0.36–2.96)	0.943				
		20 to 29	25	13 (52%)	0.95 (0.32–2.85)	0.924				
		≥30	45	24 (53%)	REF					
	Sex*	Male	61	35 (57%)	1.21 (0.69–2.11)	0.505				
		Female	74	39 (53%)	REF					
	Relation to index	Sibling	47	33 (70%)	3.83 (1.3–11.27)	0.015	3.83 (1.3–11.27)	0.015	3.25 (1.17–9.05)	0.024
		Parent	48	27 (56%)	2.09 (0.71–6.11)	0.178	2.09 (0.71–6.11)	0.178	1.8 (0.69–4.67)	0.226
		Other	42	16 (38%)	REF		REF		REF	
	Share room with index	No	58	29 (50%)	REF					
		Yes	79	47 (59%)	1.47 (0.62–3.49)	0.383				
**Index case characteristics**										
	Age (months)	6 to 17	30	20 (67%)	1.41 (0.47–4.24)	0.54				
		≥18	105	56 (53%)	REF					
	Vomiting	No	38	14 (37%)	REF		REF		REF	
		Yes	99	62 (63%)	2.87 (0.82–10.1)	0.099	2.5 (0.9–6.94)	0.079	2.42 (0.76–7.71)	0.135
	Vomiting episodes	0 to 1	84	42 (50%)	REF					
		Multiple	53	34 (64%)	1.34 (0.76–2.36)	0.315				
	Duration of diarrhea (days)	1	22	8 (36%)	REF					
		2	53	29 (55%)	0.34 (0.03–3.84)	0.381				
		≥3	62	39 (63%)	0.71 (0.29–1.75)	0.46				
	Vesikari score	4 to 9	61	26 (43%)	REF					
		10 to 14	76	50 (66%)	2.58 (0.86–7.65)	0.086				
	Cycle threshold	<15	95	59 (62%)	REF		REF			
		15+	42	17 (40%)	0.41 (0.12–1.48)	0.177	0.49 (0.17–1.41)	0.187		
	Rotavirus vaccine	No	13	7 (54%)	REF					
		≥1	124	69 (56%)	1.07 (0.26–4.45)	0.92				
		2	100	59 (59%)	1.23 (0.31–4.84)	0.764				
**Household characteristics**										
	Total residents	2 to 3	54	30 (56%)	1.3 (0.34–5.06)	0.7				
		4	36	23 (64%)	1.85 (0.38–8.96)	0.447				
		≥5	47	23 (49%)	REF					
	Other children	0	20	7 (35%)	0.46 (0.09–2.46)	0.37				
		1	59	38 (64%)	1.58 (0.45–5.49)	0.475				
		≥2	58	31 (53%)	REF					

Odds ratios, 95% confidence intervals and Wald-test p values are given for (a) univariate models, multivariate models of (b) other case *or* contact characteristics and (c) other case *and* contact characteristics.

Regarding susceptibility, infection attack rates were higher amongst siblings (70%; OR = 3.83, p = 0.015) and parents (56%; OR = 2.09, p = 0.17) compared to other household members (e.g. aunts/uncles/grandparents; 38%).

The higher risk of infection in siblings remained significant when controlling for other case and contact variables in multivariable models; greater transmissibility associated with vomiting was of borderline significance when accounting for other index case characteristics (OR = 2.50, p = 0.079) as well as in the full models accounting for contact characteristics (OR = 2.42, p = 0.135).

### Disease

There were no episodes of diarrhea reported in the healthy control households in the 10 days before enrolment. Five diarrhea episodes were reported in diarrhea control households (attack rate = 4%), all of which occurred within 3 days of onset in the index case. Twenty acute gastroenteritis (AGE) episodes were reported in case households (attack rate = 15%), six of which had times of onset 5 to 1 days prior to onset in the index case and 14 with onset 1 to 5 days after onset in the index case (Figure 2; Table 2). Only these 14 cases were considered as possible secondary episodes for subsequent analyses. 8 of the 14 possible secondary episodes were positive for rotavirus by RT-qPCR. All of the cases with onset prior to the index case were amongst older individuals (aged 5 to 37 years).

**Table 2:** Disease attack rates* amongst household contacts by their own characteristics and index case characteristics.

					Univariate		Multivariate(other case or contact characteristics)		Multivariate(case and contact characteristics)	
			n	Disease* (%)	OR (95% CI)	p	OR (95% CI)	p	OR (95% CI)	p
**Contact characteristics**										
	Age (years)*	0 to 9	26	8 (31%)	4.44 (1.78–11)	0.001	7.96 (2.97–21.3)	<.001	19.7 (2.23–173.6)	0.01
		10 to 19	64	2 (3%)	0.63 (0.26–1.52)	0.299				
		20 to 29	25	0 (0%)	…					
		≥30	44	4 (9%)	REF		REF		REF	
	Sex*	Male	58	8 (14%)	1.76 (0.54–5.79)	0.352				
		Female	72	6 (8%)	REF					
	Relation to index	Sibling	43	6 (14%)	1.17 (0.46–3.06)	0.753				
		Parent	47	3 (6%)	0.49 (0.17–1.39)	0.182				
		Other	41	5 (12%)	REF					
	Share room with index	No	56	1 (2%)	REF		REF			
		Yes	75	13 (17%)	11.5 (0.93–141.6)	0.056	15.67 (1.23–198)	0.034	43.8 (0.3–6297)	0.14
**Index case characteristics**										
	Age (months)	6 to 17	25	12 (48%)	48 (8.53–394)	<.001	45.7 (6.83–306.4)	<.001	92 (4.99–1696)	0
		≥18	106	2 (2%)	REF		REF		REF	
	Vomiting	No	38	1 (3%)	REF					
		Yes	93	13 (14%)	6.01 (0.46–78.4)	0.171				
	Vomiting episodes	0 to 1	79	2 (3%)	REF		REF		REF	
		Multiple	52	12 (23%)	11.6 (1.25–106)	0.031	3.28 (1.31–8.21)	0.011	3.63 (1.23–10.7)	0.02
	Duration of diarrhea (days)	1	22	0 (0%)	…					
		2	48	2 (4%)	0.17 (0.02–1.54)	0.118				
		≥3	61	12 (20%)	REF					
	Vesikari score	4 to 9	60	0 (0%)	REF					
		10 to 14	71	14 (20%)	…	<0.001				
	Cycle threshold	<15	93	14 (15%)	REF					
		15+	38	0 (0%)	…	0.011				
	Rotavirus vaccine	No	12	1 (8%)	REF					
		≥1	97	13 (13%)	1.7 (0.13–22)	0.68				
		2	119	13 (11%)	1.34 (0.1–17.4)	0.819				
**Household characteristics**										
	Total residents	2 to 3	51	2 (4%)	0.14 (0.01–1.46)	0.1				
		4	33	1 (3%)	0.1 (0.01–1.58)	0.102				
		≥5	47	11 (23%)	REF					
	Other children	0	19	0 (0%)	…					
		1	57	3 (5%)	0.22 (0.02–2.05)	0.185				
		≥2	55	11 (20%)	REF					

* There was not sufficient power to fit regression model when using the definition of disease of symptomatic and RT-qPCR positive, as only 8 contacts met this definition. However, the simple frequency tabulations with this outcome were consistent with the disease (regardless of test result) outcome analysis. Attack rates were higher from children who were younger (24% (6/25) from children <18 months compared to 2% (2/106) from children ≥18 months), had multiple episodes of vomiting (15% (8/82) compared to 0% from children with 1 or no episodes of vomiting (0/79)) and amongst contacts aged less than 10 years (27% (7/26) compared to contacts aged 10 years or older (0%; 0/91)).

Odds ratios, 95% confidence intervals and Wald-test p values are given for (a) univariate models, multivariate models of (b) other case *or* contact characteristics and (c) other case *and* contact characteristics.

The effects of index case characteristics (i.e. transmissibility) (i.e. index case characteristics) and contact characteristics (i.e susceptibility) on disease attack rates are shown in Table 2. Regarding transmissibility, disease attack rates were higher from children who were younger (48% from children <18 months compared to 2% from children ≥18 months) or had multiple episodes of vomiting (23% compared to 3% from children with 1 or no episodes of vomiting; OR = 11.6, p = 0.031) (Figure 3).

Regarding susceptibility to disease, attack rates were higher amongst contacts aged below 10 years (31%; OR = 4.44, p = 0.001 compared to contact aged 30 years or older) and those who shared a room with the index case (17% compared with 2%; OR = 11.5, p = 0.056).

There were only two instances where a household contact was younger than the index case. Both of these children were infected and one was symptomatic.

In the final disease model that controlled for case and contact characteristics, children <18 months were more infectious (OR = 92, p<0.001), multiple vomiting episodes were associated with high transmission risk (OR = 3.63; p = 0.02) and children aged <10 years were most susceptible to disease (OR = 19.7, p = 0.01).

### Transmission from Asymptomatically Infected Individuals?

**Figure 4:**
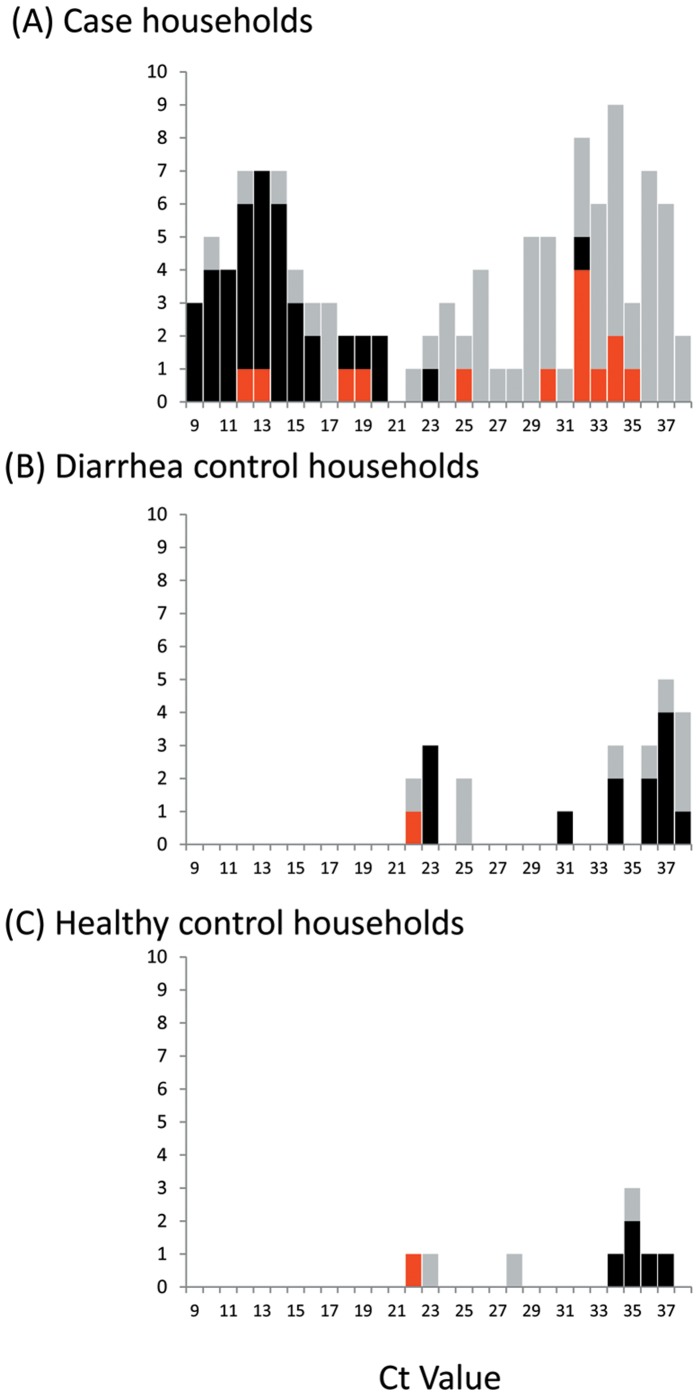
Distribution of cycle threshold (Ct) values in (A) case households (B) diarrhea control households and (C) healthy control households. Black bars indicate index children (who were positive by ELISA in the case households and negative by ELISA in the control households, by definition. Red bars indicate symptomatic household contacts; grey bars indicate asymptomatic household contacts.

Rotavirus was detected by RT-qPCR in stool from 33% (13/40) of diarrhea controls and 12% (5/40) healthy controls, all of whom were negative by EIA. Infection attack rates were 16% (7 out of 43 household members) in the diarrhea control households and 0% (out of 20 household members) in infected healthy control households. None of the household members reported diarrhea in the 10 days prior to the onset in the index healthy control, but there were 3 symptomatic cases after onset in the index diarrhea control. The Ct value of the diarrhea control was >34 in these three instances (Figure 4). Further, rotavirus was not detected in any of these three symptomatic contacts cases, suggesting that the cause of diarrhea in both the index diarrhea control and the household member was not rotavirus.

### Genotyping

We were able to determine genotypes from 36 of the 39 index cases. G2P [4] genotype was detected from 16 (44%) of these, and G2P [8] and G4P [4] was detected in one sample each. G9P was detected from 14 specimens, 12 of which had a P-type P [8], one with P [4] and one could not be P-typed. Four specimens were G3, two typed with P [8], and one each with P [4] and P [6].

**Figure 5:**
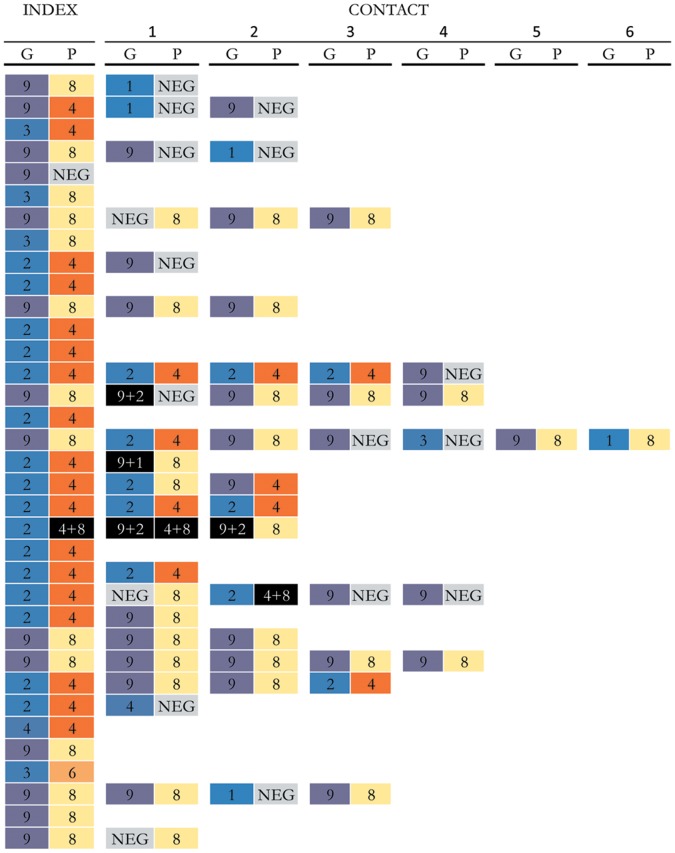
Genotype (G- and P-type) profiles of infections amongst rotavirus index cases and household contacts. The first 2 columns represent the G and P types (respectively) of the typeable index cases (n = 35) and, with each typeable household contact (n = 57) shown to the right of the index case. Single G and P-tpye infections are color-coded; mixed infections are in black and un-typeable G- or P-types are in grey.

Genotyping data were available from 52 contacts in 22 case households where the genotype of the index case was also determined (Figure 5). 38 (73%) of the 52 contacts shared a G-type or P-type with the index case. However, in only seven of the 22 households (32%) was a single genotype found amongst the index case and all household contacts. In some households, there was a single household contact with a different genotype than the index case. For example, in one household the index case was G9P [8], along with 2 household contacts, while another household contact was a G1. Still in other households, there was clear evidence of circulation of multiple viruses. For example, in one household, G2P [4] was detected in the index case, G9P [8] in one contact with both viruses in another household member. In addition, P [8] was detected in the index case and G [2] was detected in the G9P [8]-infected contact, suggesting that all three individuals were infected with both viruses.

A total of 10 G1 viruses were detected (1 healthy control, 1 diarrhea control, 2 household contact of diarrhea controls and 7 household contacts of cases). None were Rotarix vaccine strain.

## Discussion

Our results suggest that rotavirus is highly transmissible in household settings. Although secondary cases of disease are rare among adults, transmission of virus resulting in asymptomatic infection is exceedingly common. Overall, 55% of household contacts showed evidence of infection. Disease attack rates were approximately 30% amongst children under the age of ten years. Moreover, we have identified factors associated with transmissibility in cases (frequent vomiting/severity and being under 18 months of age) as well as susceptibility in contacts (being a sibling of or sharing a room with the index case). Infection rates were consistently high across all ages, whereas young age was a risk factor only for disease susceptibility. RT-qPCR was vital for detection of asymptomatic infection.

Our inferences are strengthened by the recruitment of two groups of control households. First, we are able to confirm that presence of rotavirus is relatively rare among household members where the index child did not have rotavirus. Thus, the high prevalence of infection detected in case households cannot be explained by background levels of infection. Moreover, the data support the notion that viral load and, therefore EIA diagnostic results, are strongly associated with symptomatic disease [11]. Nearly all asymptomatic household members in case, diarrhea control and health control households had a Ct value >20.

The fact that we did not detect rotavirus in stools from contacts of asymptomatically infected healthy controls suggests that symptoms are crucial for transmission of rotavirus. Interestingly, rotavirus was detected in 13% of contacts of diarrhea controls who were found to have low-level rotavirus infections (high Ct values). This suggests that rotavirus can be transmitted, even if it is not the cause of disease, when the index case is symptomatic from another cause. However, we found no instances of asymptomatic infection leading to transmission resulting in symptomatic disease. To the contrary, we found that symptoms, specifically vomiting, are associated with transmission of infection and disease.

There are at least two important limitations of our study. First, we cannot be certain that the child who presented to the clinic for diarrhea was the first to be infected in the household, so we have been careful to refer to these children as index, rather than primary, cases. In most situations, the index child likely was the primary case, but amongst the 20 symptomatic episodes amongst household contact, 6 occurred prior to the index case. These 6 individuals were excluded from disease risk factor analysis. However, because we found no evidence of true asymptomatic transmission, it is highly likely that the symptomatic index cases transmitted virus to the large number of asymptomatic but infected household contacts, rather than the reverse. A second important limitation relates to the possibility of cross-contamination of specimens within the household. Because of the timing of collection and the need to get a stool from all members of a household, collection was performed by individuals themselves or a responsible adult in the case of young children. Despite clear instructions on specimen collection by trained study nurses, there remains the possibility of contamination in the household setting. While we cannot rule out that some detections of virus could be results of contamination, the elucidation of factors related to disease transmission (relationship to index, sharing a room with index, vomiting in index) argue for a genuine pattern consistent with transmission, rather than random contamination.

Previous studies of rotavirus household transmission have not used genotyping data to aid in the interpretation of apparent transmission links. We hypothesized that the same virus would be detected amongst all infected household members, and thereby strengthen evidence of a transmission event. Instead, the genotyping results presented a more complicated picture. In some households, a single genotype was in fact detected amongst all infected individuals. In others, there was evidence of multiple rotavirus genotypes infecting household members. In others still, there was evidence to suggest that there was more than one introduction into the household (based on multiple viruses, but no co-infections). Apparent co-infections were more common amongst household contacts than cases. This could either be because asymptomatically infected individuals are more likely to harbor multiple rotavirus types, [18] or, that PCR-based detection methods are more likely to pick up only the most abundant virus especially in acute pediatric infections where viral load may be several orders of magnitude higher for the virus actually causing disease. Therefore, it may be that both symptomatic children and their asymptomatic contacts have the same diversity of viruses in their stools, but a single virus is more likely to be detected in robust childhood infections.

Our results would suggest that either prevention of infection or severe symptoms through vaccination could also indirectly avert secondary household or community cases. Unfortunately, we were unable to directly assess the effect of vaccination on transmissibility since >85% of the age-eligible population was vaccinated.

In conclusion, these results highlight the remarkable infectiousness of rotavirus, and the pathways through which transmission occurs within household settings. In the future, household transmission studies should be implemented during the roll-out of rotavirus vaccines or in partially-vaccinated populations, in order to be adequately powered to quantify the impact of vaccination on transmission.

## Supporting Information

Files S1 **Accession numbers for submitted strains.** Strain names and accession numbers for all new viral sequence data deposited in GenBank.
(PDF)
