# Correction: Household Transmission of Rotavirus in a Community with Rotavirus Vaccination in Quininde, Ecuador

**DOI:** 10.1371/annotation/b1efd2d1-cc25-44c8-99e1-4bcdd3b8ccc8

**Published:** 2013-09-24

**Authors:** Ben Lopman, Yosselin Vicuña, Fabian Salazar, Nely Broncano, Matthew D. Esona, Carlos Sandoval, Nicole Gregoricus, Michael D. Bowen, Daniel Payne, Martiza Vaca, Martha Chico, Umesh Parashar, Philip J. Cooper

A funding organization and grant were incorrectly omitted from the Funding Statement. The following sentence should be included in the Funding Statement:

"The collection of data in the field was supported also by Wellcome Trust grant 088862/Z/09/Z." 

