# Supplementary material for: Household Transmission of Rotavirus in a Community with Rotavirus Vaccination in Quininde, Ecuador
Source: PLoS One. 2013 Jul 9;8(7):e67763. doi: 10.1371/journal.pone.0067763 (PMC3706538; doi:10.1371/journal.pone.0067763)
Supplement: Files S1 — Accession numbers for submitted strains. Strain names and accession numbers for all new viral sequence data deposited in GenBank. (PDF) [file pone.0067763.s001.pdf]

VP7: G2 strains

**Strain name**	**Accession #**
RVA/Human-wt/ECU/2012826166/2011/G2P[8]	KC951932
RVA/Human-wt/ECU/2012826168/2011/G2P[4]	KC951933
RVA/Human-wt/ECU/2012826169/2011/G2P[4]	KC951934
RVA/Human-wt/ECU/2012826186/2011/G2P[4]	KC951935
RVA/Human-wt/ECU/2012826189/2011/G2P[4]	KC951936
RVA/Human-wt/ECU/2012826191/2011/G2P[4]	KC951937
RVA/Human-wt/ECU/2012826192/2011/G2P[4]	KC951938
RVA/Human-wt/ECU/2012826196/2011/G2P[4]	KC951939
RVA/Human-wt/ECU/2012826197/2011/G2P[4]	KC951940
RVA/Human-wt/ECU/2012826198/2011/G2P[4]	KC951941
RVA/Human-wt/ECU/2012826214/2011/G2P[4]	KC951942
RVA/Human-wt/ECU/2012826225/2012/G2P[4]	KC951943
RVA/Human-wt/ECU/2012826228/2012/G2P[4]	KC951944
RVA/Human-wt/ECU/2012826190/2011/G2P[4]	KC951945

VP7: G9 strains

Strain name	Accession #
RVA/Human-wt/ECU/2012826188/2011/G9P[4]	KC951946
RVA/Human-wt/ECU/2012826193/2011/G9P[8]	KC951947
RVA/Human-wt/ECU/2012826200/2012/G9P[8]	KC951948
RVA/Human-wt/ECU/2012826213/2011/G9P[8]	KC951949
RVA/Human-wt/ECU/2012826217/2011/G9P[8]	KC951950
RVA/Human-wt/ECU/2012826218/2011/G9P[8]	KC951951
RVA/Human-wt/ECU/2012826219/2011/G9P[8]	KC951952
RVA/Human-wt/ECU/2012826220/2011/G9P[8]	KC951953
RVA/Human-wt/ECU/2012826221/2012/G9P[8]	KC951954
RVA/Human-wt/ECU/2012826222/2012/G9P[8]	KC951955
RVA/Human-wt/ECU/2012826223/2012/G9P[8]	KC951956
RVA/Human-wt/ECU/2012826226/2012/G9P[8]	KC951957
RVA/Human-wt/ECU/2012826037/2011/G9P[8]	KC951958
RVA/Human-wt/ECU/2012826038/2011/G9P[8]	KC951959
RVA/Human-wt/ECU/2012826143/2011/G9P[8]	KC951960
RVA/Human-wt/ECU/2012826144/2011/G9P[8]	KC951961
RVA/Human-wt/ECU/2012826149/2011/G9P[8]	KC951962
RVA/Human-wt/ECU/2012826153/2011/G9P[8]	KC951963
RVA/Human-wt/ECU/2012826172/2011/G9P[8]	KC951964
RVA/Human-wt/ECU/2012826173/2011/G9P[8]	KC951965
RVA/Human-wt/ECU/2012826175/2011/G9P[8]	KC951966
RVA/Human-wt/ECU/2012826177/2011/G9P[8]	KC951967
RVA/Human-wt/ECU/2012826180/2011/G9P[8]	KC951968
RVA/Human-wt/ECU/2012826242/2012/G9P[8]	KC951969
RVA/Human-wt/ECU/2012826245/2012/G9P[8]	KC951970

VP4: P[4] strains

Strain name	Accession #
RVA/Human-wt/ECU/2012826168/2011/G2P[4]	KC951971
RVA/Human-wt/ECU/2012826169/2011/G2P[4]	KC951972
RVA/Human-wt/ECU/2012826186/2011/G2P[4]	KC951973
RVA/Human-wt/ECU/2012826188/2011/G9P[4]	KC951974
RVA/Human-wt/ECU/2012826189/2011/G2P[4]	KC951975
RVA/Human-wt/ECU/2012826192/2011/G2P[4]	KC951976
RVA/Human-wt/ECU/2012826196/2011/G2P[4]	KC951977
RVA/Human-wt/ECU/2012826197/2011/G2P[4]	KC951978
RVA/Human-wt/ECU/2012826198/2011/G2P[4]	KC951979
RVA/Human-wt/ECU/2012826225/2011/G2P[4]	KC951980
RVA/Human-wt/ECU/2012826228/2011/G2P[4]	KC951981

<u>VP4: P[8] strains</u>

Strain name	Accession #
RVA/Human-wt/ECU/2012826037/2011/G9P[8]	KC951982
RVA/Human-wt/ECU/2012826143/2011/G9P[8]	KC951983
RVA/Human-wt/ECU/2012826149/2011/G9P[8]	KC951984
RVA/Human-wt/ECU/2012826152/2011/G9P[8]	KC951985
RVA/Human-wt/ECU/2012826166/2011/G2P[8]	KC951986
RVA/Human-wt/ECU/2012826172/2011/G9P[8]	KC951987
RVA/Human-wt/ECU/2012826173/2011/G9P[8]	KC951988
RVA/Human-wt/ECU/2012826175/2011/G9P[8]	KC951989
RVA/Human-wt/ECU/2012826200/2011/G9P[8]	KC951990
RVA/Human-wt/ECU/2012826217/2012/G9P[8]	KC951991
RVA/Human-wt/ECU/2012826220/2012/G9P[8]	KC951992
RVA/Human-wt/ECU/2012826222/2012/G9P[8]	KC951993
RVA/Human-wt/ECU/2012826242/2012/G9P[8]	KC951994
